# *H. pylori* infection is related to mitochondrial microsatellite instability in gastric carcinogenesis

**DOI:** 10.1186/s13027-016-0078-5

**Published:** 2016-07-12

**Authors:** Xianlong Ling, Haoxiang Zhang, Caifei Shen, Wu Yan, Pu Wang, Ji Feng, Zhihong Peng, Guiyong Peng, Wensheng Chen, Dianchun Fang

**Affiliations:** Department of Gastroenterology, Southwest Hospital, Third Military Medical University, Chongqing, 400038 China

**Keywords:** *H. pylori*, Mitochondrial microsatellite instability, IL-8, Gastric cancer

## Abstract

**Bachground:**

To assess the correlation of *H. pylori* infection with mitochondrial microsatellite instability (mtMSI) and IL-8 in gastric carcinogenesis.

**Methods:**

*H. pylori* infection was evaluated through histology and a urease breath test; mtMSI was measured using PCR-single strand conformation polymorphism (PCR-SSCP); IL-8 was analyzed with ELISA methods.

**Results:**

The detection rate of mtMSI was significantly higher in specimens with *H. pylori* infection than in those without *H. pylori* infection (*P* < 0.05). The levels of IL-8 were significantly higher in specimens with mtMSI than in those without mtMSI (*P* < 0.01).An association of mtMSI with the intestinal histological type was found (*P* < 0.05). Increased IL-8 levels induced by *H. pylori* were related to the invasion, lymphnode spreading and clinical stage of gastric cancer (*P* < 0.05).

**Conclusions:**

*H. pylori* infection is related to mitochondrial microsatellite instability in the early steps of gastric cancer development. IL-8 may play a role in the development of mtMSI induced by *H. pylori*. Our results support a role for mtMSI in different mechanisms of gastric carcinogenesis.

## Background

Gastric carcinogenesis is a complex multistep multifactorial event, in which the role of *Helicobacter pylori* (*H. pylori*) infection, the major etiopathogenic factor underlying chronic antral gastritis and duodenal ulcer, has been established in recent years [1, 2]. Infection with *H. pylori*, especially with CagA+ strains, has been associated with an increased risk of noncardia gastric adenocarcinoma [3, 4]. The epidemiological link between *H. pylori* and gastric carcinoma has been confirmed, and the rate of gastric carcinoma development in the Japanese population with *H. pylori*-gastritis was reported to be 2.9 % during a 7.8-year period [5]. The mechanism by which *H. pylori* contributes to gastric carcinogenesis is still largely unknown. The chronic inflammatory reaction caused by the bacterium is directly involved in gastric carcinogenesis through its potential to cause excess production of reactive oxygen species and consequent mutagenic and carcinogenic changes in DNA [6, 7]. ROS production has been shown to occur in association with this bacterium both in vitro and in vivo [8–10]. The dietary intake of antioxidants, measured as the total antioxidant potential, is inversely associated with the risk of both cardia and distal cancer [11].

*H. pylori* infection results in the induction of a number of genes in host cells that are potential determinants of inflammation. Interleukin-8 (IL-8), a CXC chemokine specific for neutrophil granulocyte chemotaxis, is a central mediator of the inflammatory response to *H. pylori* and has been found to be involved in *H. pylori*-associated gastric carcinogenesis [12]. IL-8 induction in gastric epithelial cells has been clearly correlated with a functional *cagA* gene [13]. In *H. pylori* strains that express CagA, cytokine expression has been linked to an elevated inflammatory response *in vivo* [14]. The expression of IL-8 was found to be 10 times higher in gastric cancer tissue than in normal tissue and to be two times higher in advanced gastric cancer tissue than early cancer tissue [15]. The eradication of *H. pylori* decreases IL-8 expression significantly, suggesting that gastric cancer may be associated with the inflammatory process in the gastric mucosa through the over-expression of IL-8 [16]. It is therefore expected that intervention with an IL-8 inhibitor would inhibit or reverse the process of *H. pylori*-related carcinogenesis.

Genetic instability plays a key role in neoplastic transformation and progression [17, 18]. A previous study by our group showed that in gastric carcinomas, such genetic instability may be classified into two different forms, in which hypermutability occurs by means of either chromosomal instability or microsatellite instability (MSI) [19, 20]. Although a wide spectrum of MSI alterations in the nuclear DNA (nMSI) of cancer cells has been established, little attention has been paid to MSI in the mitochondrial DNA (mtMSI) of *H. pylori*-infected gastric tissues. The mitochondrial genome is more vulnerable to oxidative damage and experiences a higher rate of mutation than the nuclear genome [21]. The biology of the mitochondrion suggests that its genome may be an attractive target for ROS, which could drive tumorigenesis. Tumor formation is often associated with mtDNA mutations and alterations in mitochondrial genome function. Our previous studies have identified sequential accumulation of mtMSI in the histological progression from chronic gastritis to gastric cancer, suggesting that mtMSI may play an early and important role in the gastric carcinogenesis pathway, especially in intestinal-type cancer and distal gastric cancer [22]. However, the role of mtMSI following oxidative DNA damage in *H. pylori*-related gastric carcinoma is less clear. In the present study, we explored the associations between *H. pylori* infection, mtMSI and IL-8 to elucidate whether mtMSI triggers the progression from *H. pylori*-gastritis to intestinal metaplasia and dysplasia and, finally, to gastric cancer.

## Methods

### Tissue samples

A total of 122 fresh samples from surgically resected gastric carcinomas and 100 fresh samples of endoscopically obtained non-neoplastic gastric mucosa (40 chronic gastritis, 40 intestinal metaplasia and 20 dysplasia mucosa) were studied. In addition, we collected 30 gastric biopsies from patients without any gastric disease as a control for the analysis of mtMSI and IL-8 alterations. Hematoxylin-eosin (HE) staining was performed for histopathological diagnosis and to evaluate gastritis, atrophy, intestinal metaplasia, dysplasia and cancer. Genomic DNA was isolated using standard proteinase K digestion and phenol-chloroform extraction protocols. No patient had received non-steroidal anti-inflammatory drugs, proton pump inhibitors, or antibiotics within the previous three months. None of the gastric cancer patients included in the present series had a family history suggestive of HNPCC or had received chemotherapy or radiation therapy. This study was approved by the Bioethical Committee of the southwest hospital, and all patients signed informed consent forms before inclusion.

### Histology

The surgically resected and biopsy specimens were fixed in 10 % buffered formalin, embedded in paraffin wax, cut into sequential 4 μm sections, and subjected to hematoxylin and eosin (H&E) and modified Giemsa staining. In most cases, we cut three to five serial sections from each specimen, and multiple high power fields were examined. Virtually all of the specimens included the surface epithelium and muscularis mucosa. The specimens were examined by one experienced histologist blinded to the patient’s clinical diagnosis and *H. pylori* characteristics*.*

### *H. pylori* assessment

The presence of *H. pylori* was determined through histology and a urease breath test. Patients were classified as *H. pylori-*positive if at least one of the examinations produced a positive result.

### IL-8 quantification

For IL-8 protein quantification, we used the same biopsy specimens as were used for *H. pylori* detection. Biopsy specimens were placed in 1.5 ml of PBS (pH 7.4) and were immediately homogenized using a tissue homogenizer, as described previously [23]. Aliquots of the homogenate supernatant, obtained via centrifugation (10,000  *g* for 10 min), were stored at −80 °C until the assessment of total proteins using a modified Lowry method. IL-8 in the biopsy homogenate supernatant was measured via ELISA using commercially available assay kits (Research and Diagnostic Systems, Minneapolis, Minnesota, USA). The assays were performed in duplicate according to the manufacturer’s instructions. In our laboratory, the ELISA's sensitivity for IL-8 was approximately 10 pg/ml.

### mtMSI detection

PCR-single-strand conformation polymorphism (PCR-SSCP) analysis was performed to amplify mtDNA microsatellite sequences using published primers [24]. The primers consisted of 2 D-loop regions and 5 coding regions (Table 1). The reaction conditions and procedures were similar to those reported by Hebano et al. [24].

**Table 1:** Primer sequences for PCR analysis

Repeat sequence	mtDNA region	Position	Annealing(°C)	Primer(5’-3’)
(C)n	270-425	D-loop	58	TCCACACAGACATCAATAACA
				AAAGTGCATACCGCCAAAAG
(CA)n	467-556	D-loop	55	CCCATACTACTAATCTCATCAA
				TTTGGTTGGTTCGGGGTATG
(C)6	3529-3617	ND1	55	CCGACCTTAGCTCTCACCAT
				AATAGGAGGCCTAGGTTGAG
(A)7	4555-4644	ND2	55	CCTGAGTAGGCCTAGAAATAAA
				ACTTGATGGCAGCTTCTGTG
(T)7	9431-9526	C0III	55	CCAAAAAGGCCTTCGATACG
				GCTAGGCTGGAGTGGTAAAA
(C)6 and(A)8	12360-12465	ND5	55	CACCCTAACCCTGACTTCC
				GGTGGATGCGACAATGGATT
(CCT)3 and(AGC)3	12940-13032	ND5	55	GCCCTTCTAAACGCTAATCC
				TCAGGGGTGGAGACCTAATT

Each PCR product was digested with the appropriate restriction enzymes and electrophoresed at 300 V at 22 °C for 2 h in a 7.5 % polyacrylamide gel containing 50 mmol/L boric acid, 1 mmol/L EDTA and 2.5 % glycerol. After silver staining, PCR products that showed a mobility shift were defined as showing mtMSI. All analyses were repeated twice to rule out PCR artifacts.

### Statistical analysis

The mucosal level of the IL-8 protein was expressed in pg/mg biopsy protein, and the data were expressed as the median and range. Wilcoxon’s matched pairs test was used to compare differences in IL-8 protein expression between *H. pylori-*positive and *H. pylori-*negative specimens. The relationship between *H. pylori* and the production of IL-8 and mtMSI was assessed using Spearman’s rank correlation coefficient and the Chi-square test with Yates’ correction. A *P* value of less than 0.05 was accepted as statistically significant.

## Results

### *H. pylori* infection in different gastric diseases

As shown in Table 2, the rate of *H. pylori* infection was 45.0 % in chronic gastritis, 55.0 % in intestinal metaplasia, 45.0 % in dysplasia and 42.6 % in gastric cancer. No *H. pylori* was detected in normal gastric mucosae. The rates of *H. pylori* infection in chronic gastritis, intestinal metaplasia, dysplasia and gastric cancer were significantly higher than in the normal gastric mucosa.

**Table 2:** *H. pylori* infection in different gastric diseases

	*n*	*H. pylori*+	*H. pylori* -
Normal mucosa	30	0	30
Chronic gastritis	40	18(45.0)	22(65.0)
Intestinal metaplasia	40	22(55.0)	18(45.0)
Dysplasia	20	9(45.0)	11(55.0)
Gastric cancer	122	52(42.6)	70(57.4)

### *H. pylori* infection and IL-8 expression

**Fig. 1:**
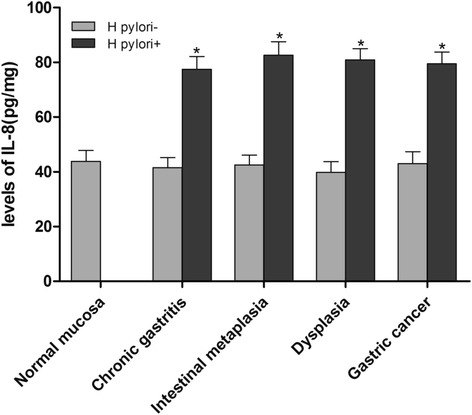
The relationship between *H. pylori* infection and IL-8 expression. The levels of IL-8 were significantly higher in various gastric mucosae with *H. pylori* infection than in those without *H. pylori* infection (*P < 0.01)

The IL-8 expression levels associated with different gastric pathologies are shown in Fig. 1. *H. pylori* infection was associated with increased expression of IL-8; the level of IL-8 was significantly higher in various gastric mucosae with *H. pylori* infection than in those without *H. pylori* infection (*P* < 0.01).

### *H. pylori* infection and mtMSI

**Fig. 2:**
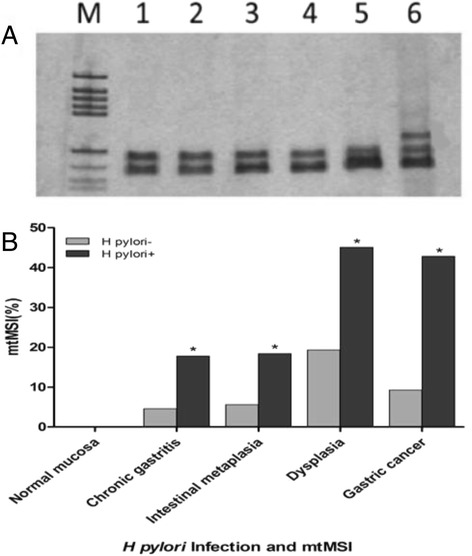
The relationship between *H. pylori* infection and mtMSI. **a**. Detection of mtMSI via PCR-single strand conformation polymorphism analysis. Lane 6 shows conformational variants associated with mtMSI. **b**. The detection rate of mtMSI was significantly higher in specimens with *H. pylori* infection than in those without *H. pylori* infection (*P < 0.05)

All tumor and non-neoplastic gastric mucosal samples were screened for mtMSI at seven repeat sites using the PCR-RFLP method. Fig. 2a shows a representative mobility-shift band compared with normal counterparts. The rates of mtMSI in different gastric pathologies are shown in Fig. 2b. *H. pylori* infection was associated with increased mtMSI in various gastric pathologies, and the rate of mtMSI was significantly higher in specimens with *H. pylori* infection than in those without *H. pylori* infection (*P* < 0.01)*.*

### Expression of IL-8 in gastric mucosae with mtMSI

**Fig. 3:**
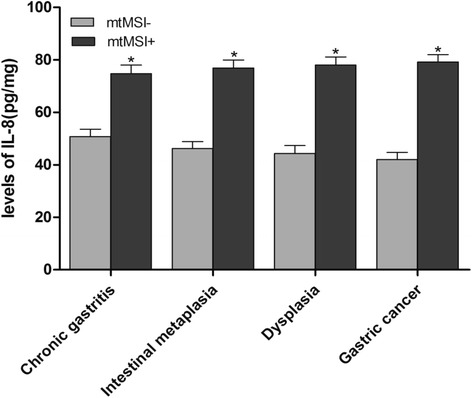
IL-8 levels in gastric mucosae with mtMSI. The levels of IL-8 were significantly higher in gastric mucosae with mtMSI than in those without mtMSI (*P < 0.05)

The relationship between IL-8 expression and mtMSI is shown in Fig. 3. The levels of IL-8 were significantly higher in gastric mucosae with mtMSI than in those without mtMSI (*P* < 0.05).

### Relationship between IL-8 levels and clinicopathological characteristics of gastric cancer

The relationships between IL-8 levels and the clinicopathological characteristics of gastric cancer are shown in Table 3. Associations between IL-8 levels and invasion, lymph node spreading and clinical staging were found (*P* < 0.01), whereas no significant correlation was found between IL-8 levels and the age at diagnosis, sex or tumor size (*P* > 0.05).

**Table 3:** Relationships between IL-8 levels and clinicopathological characteristics of gastric cancer

Characteristic		*n*	level of IL-8
Sex	Male	88	62.2 ± 3.1
	Female	34	58.2 ± 3.2
Age	<40 years	39	58.2 ± 3.6
	>40 years	83	61.2 ± 3.4
Size	<5 cm	62	57.2 ± 3.5
	>5 cm	60	63.2 ± 2.9
Histological type			
	Intestinal	72	64.2 ± 3.3
	Diffuse	50	56.2 ± 3.9
Tumor location			
	Distal	81	57.2 ± 2.8
	Proximal	41	63.2 ± 3.5
Invasion			
	Within the wall	62	48.2 ± 3.1*
	Invading serosa	60	72.2 ± 2.7
Lymph node spread			
	Absent	58	35.2 ± 2.6*
	Present	64	76.2 ± 3.1
TNM stage			
	I/II	53	38.4 ± 3.2*
	III/IV	69	74.5 ± 2.9

*P < 0.01 vs. the invading serosa, lymph node spread and stage III/IV groups

### Clinicopathological characteristics of mtMSI-positive gastric cancer

The clinicopathological characteristics of mtMSI-positive cases were compared with those of cases that were mtMSI negative (Table 4). Associations of mtMSI with the intestinal histological type and a distal location were found (*P* < 0.05), whereas no significant correlation was found between mtMSI and the age at diagnosis, sex, tumor size, tumor location, invasion, lymph node spread or clinical stage (*P* > 0.05).

**Table 4:** Clinicopathological characteristics of 122 gastric cancer patients with mtMSI

Characteristic		*n*	mtMSI+	mtMSI-
Age	<40 years	39	9	30
	>40 years	83	19	64
Sex	Male	88	20	68
	Female	34	8	18
Size	<5 cm	62	11	51
	>5 cm	60	17	43
Histological type	Intestinal	72	19*	53
	Diffuse	50	9	41
Tumor location	Distal	81	21	60
	Proximal	41	7	34
Invasion	Within the wall	62	18	44
	Invading serosa	60	10	50
Lymph node spread	Absent	58	17	41
	Present	64	11	53
TNM stage				
	I/II	53	13	40
	III/IV	69	15	54

*P < 0.05 vs. diffuse-type groups

## Discussion

Although a causal relationship exists between *H. pylori* infection and the development of gastric carcinoma, the molecular mechanisms underlying this relationship have remained elusive. In this study, we show that *H. pylori* infection may correlate with up-regulated IL-8 expression and mtMSI in the gastric mucosa of patients with various gastric pathologies. Up-regulation of IL-8 is correlated with mtMSI. These findings are consistent with IL-8 and mtMSI playing important roles in *H. pylori-*associated gastric cancer. Taking into consideration the increase in the mtMSI frequency observed in *H. pylori*-associated lesions, our results suggest early involvement and continuous accumulation of mtMSI in gastric cells with *H. pylori* infection that have entered the multistep gastric carcinogenesis pathway.

Mutations in the mitochondrial genome have been detected in nearly every type of cancer investigated to date [25], including gastric cancer and the corresponding preneoplastic lesions [26]. *H. pylori* infection has been reported as an important risk factor for gastric carcinogenesis [5, 27]. Previous studies have identified a significant association of *H. pylori* infection and gastric cancer with nMSI [24, 28]. Although some studies addressing mitochondrial mutations in gastric cancer have attempted to identify the role of mitochondrial genetic instability [22, 24, 29], reports on the role of *H. pylori* in mtMSI in gastric carcinogenesis have been rare. mtDNA mutations are associated with *H. pylori* infections responsible for chronic gastritis and peptic ulcer tissues, indicating that the consequences of *H. pylori* infection include the aggregation of mutations in mtDNA in early phases of gastric cancer development. Machado et al. [30] showed that gastric epithelial cells are closely related to the genetic instability of mitochondrial DNA due to *H. pylori* infection. To study the role of mtMSI in *H. pylori*-associated gastric carcinogenesis, we analyzed mtMSI in *H. pylori*-positive and *H. pylori*-negative gastric mucosa using seven microsatellite markers known to be altered in gastrointestinal carcinomas. It was found that as carcinogenesis progresses, the level of mtMSI increases, and the rate of mtMSI was found to be significantly higher in the *H. pylori*-positive groups than the *H. pylori*-negative groups, implying that mtMSI might play a role in the occurrence of those gastric cancers that are *H. pylori* positive.

The mechanisms underlying mtMSI induced by *H. pylori* in the gastric mucosa remain unclear. Reactive oxygen species (ROS) are commonly released in gastric mucosae that are inflamed as a result of infection with *H. pylori*, especially with CagA+ strains, and could be responsible for mtMSI-positive gastric cancer [31, 32]. The mitochondrial genome is particularly susceptible to oxidative damage and mutation because of the high rate of ROS generation in this organelle and its inefficient DNA repair system [33, 34]. Increased damage caused by ROS and defective DNA repair are the two causes that have been proposed to explain mtMSI in *H. pylori*-associated gastric cancer [31, 33]. A significant correlation between high IL-8 expression in the gastric mucosa and gastric cancer risk has been reported [35]. In IL-8 transgenic mice, the expression of IL-8 increases tumorigenesis, suggesting that IL-8 might play a crucial role in gastrointestinal cancers [36]. The induction of higher levels IL-8 expression by *H. pylori* has been observed in gastric carcinoma and premalignant lesions [12]. In the present study, we found that the levels of IL-8 were significantly higher in patients with mtMSI than in those without mtMSI, suggesting that IL-8 may play a role in the development of mtMSI induced by *H. pylori*. However, the mechanisms through which IL-8 leads to the development of mtMSI in *H. pylori*-positive gastric mucosae need to be studied further.

Cancers arising from different mutational pathways are thought to have different clinical features. nMSI+ gastric cancer is characterized by an older age, antral location, intestinal type, lower prevalence of lymph node metastasis, and a lower pTNM stage [37, 38]. However, the clinicopathological characteristics of mtMSI+ gastric cancers remain unclear. In the current study, we did not find an obvious relationship between mtMSI and tumor size, the depth of invasion, node metastasis or clinical stage, indicating a limited role of mtMSI in predicting the prognosis of gastric carcinoma. However, a marked difference in mtMSI was noted in gastric cancers distinguished by histological type. mtMSI was significantly more frequent in intestinal-type gastric cancers than diffuse-type gastric cancers, suggesting that mtMSI is a predisposing event in intestinal gastric cancer.

IL-8 can regulate neovascularization, thereby promoting the growth and spread of human gastric carcinoma [39]. Yamaoka et al. found that the expression of IL-8 was 10 times higher in gastric cancer tissue than in normal tissue and it was twice as high in advanced gastric cancer tissue compared with early cancer tissue [15]. Macrì et al. [40] reported that serum levels of IL-8 serve as a marker of gastric cancer. Increased expression of IL-8 mRNA in tissue extracts from gastric cancer patients has been associated with certain clinicopathological aspects of the disease, including a poor prognosis [41]. In a previous study, we showed that co-culture with *H. pylori* stimulates AGS cell motility and invasion, upregulates ezrin expression at the protein level and induces a Hummingbird phenotype [42]. In the present study, IL-8 levels were found to be associated with invasion, lymph node spreading and clinical stage. These observations indicate that high levels of IL-8 may be associated with a poor prognosis and that IL-8 may be indicative of more aggressive gastric cancer.

## Conclusion

In conclusion, we have demonstrated that mtMSI is an early and important event in the progression of gastric carcinogenesis, especially in intestinal-type gastric cancer. *H. pylori* infection contributes to mtMSI in early steps of gastric cancer development. IL-8 may play a role in the development of mtMSI induced by *H. pylori*. Our results support a role for mtMSI in different mechanisms of gastric carcinogenesis. Because the majority of patients with *H. pylori* infection will not progress to cancer and only a subset of these patients harbor mtMSI, it is conceivable that patients with *H. pylori* infection displaying mtMSI are at greater risk of developing gastric cancer than those without instability. As a surrogate marker for the risk of gastric cancer development, the role of mtMSI in gastric lesions with *H. pylori* infection warrants further investigation.

## Abbreviations

*H. pylori*, *Helicobacter pylori*; HE, Hematoxylin-eosin; IL-8, Interleukin-8; mtMSI, mitochondrial microsatellite instability; PCR-SSCP, PCR-single strand conformation polymorphism.
